# On the mechanism of the electrophysiological changes and membrane lesions induced by asbestos fiber exposure in *Xenopus laevis* oocytes

**DOI:** 10.1038/s41598-019-38591-x

**Published:** 2019-02-14

**Authors:** Annalisa Bernareggi, Giorgia Conte, Andrew Constanti, Violetta Borelli, Francesca Vita, Giuliano Zabucchi

**Affiliations:** 1 Department of Life Sciences and Centre for Neuroscience B.R.A.I.N., University of Trieste, via Fleming 22, 34127 Trieste, Italy; 2 Department of Pharmacology, UCL School of Pharmacy, 29/39 Brunswick Square, London, WC1N 1AX United Kingdom; 3 Department of Life Sciences, University of Trieste, via Valerio 28/1, 34127 Trieste, Italy

## Abstract

The so-called amphibole asbestos fibers are enriched with mineral iron ions, able to stimulate ROS production. We recently reported that crocidolite asbestos was able to interact with the cell membranes of *Xenopus laevis* oocytes, to alter their electrical membrane properties. Here, we found that applied iron ions (Fe^3+^) or H_2_O_2_ (for ROS generation) mimicked these effects, suggesting that at least one effect of iron-containing asbestos fiber exposure was mediated by ROS production. Furthermore, combined Fe^3+^ and H_2_O_2_ acted synergistically, producing a membrane effect stronger than that induced by these factors alone. Similar to crocidolite, these changes peaked within 30 minutes of incubation and vanished almost completely after 120 min. However, in the presence of cytochalasin D, which inhibits membrane actin repair mechanisms, crocidolite or applied Fe^3+^/H_2_O_2_ invariably produced oocyte cell death. While the electrophysiological modifications induced by crocidolite suggested a modification of an intrinsic chloride ion channel, the morphological appearance of the treated oocytes also indicated the formation of membrane “pores”; the effects of asbestos exposure may therefore consist of multiple (not necessarily exclusive) underlying mechanisms. In conclusion, using *Xenopus* oocytes allowed us for the first time, to focus on a specific membrane effect of crocidolite asbestos exposure, which deserves to be tested also on human lung cell lines. Much available evidence suggests that asbestos fibers damage cells through the production of ROS. Our present data confirm that crocidolite fibers can indeed trigger ROS-mediated damaging effects in the oocyte cell membrane, provided iron ions and H_2_O_2_ are available for ROS production.

## Introduction

Asbestos is a very dangerous fibrous silicate mineral whose inhalation can lead to chronic lung inflammation and aggressive lung and pleural tumors. Various studies have suggested that fiber dimension, surface properties, and physical durability are important criteria for the carcinogenicity of the fibers^1^. However, despite the well-known toxicity of asbestos, the mechanism of interaction between asbestos fibers and biological cell membranes is still incompletely understood. Considering that these molecular mechanisms are those that allow the fibers to enter the target cell cytosol and then the nuclear compartment to interfere with the DNA integrity and transcriptional activity, their knowledge is compelling in helping to find new therapeutic approaches for treating exposed subjects. We recently described *Xenopus laevis* oocytes as a suitable model for studying in detail, the interaction between asbestos fibers and biological cell membranes at the electrophysiological and morphological level^2^. We found that exposure of the oocyte cells to aqueous suspensions of amosite (brown asbestos) or crocidolite (blue asbestos), significantly affected their electrical membrane properties as well as the morphology of the cells, and proposed that the fibers, either by adsorbing onto the cell surface and/or traversing the membrane, somehow created a “pore” through which ion fluxes (most likely Cl^−^) could occur to change the resting membrane potential and membrane resistance of the cells. Alternatively, our results could also be explained by a surface activation/modulation of an ion channel(s) already present in the oocyte membrane by asbestos, in order to alter its permeability characteristics. How these observed permeability and structural changes in *Xenopus* oocytes are related to asbestos toxicity in mammalian cells is presently unclear. Among the possible mechanisms responsible for asbestos-induced cell damage, there is reactive oxygen species (ROS) production, and thus the creation of a cellular oxidative stress^3,4^. So-called amphibole fibers (crocidolite, amosite, tremolite, anthophyllite, and actinolite) are reported to stimulate the production of ROS in two different ways: through the catalytic presence of Fe^2+^ and Fe^3+^ on the surface of the asbestos fibers (Fenton and Haber Weiss reactions), or by activation of phagocytic cells^5^. In the present study, we investigated in detail, the possible involvement of Fe^2+^/Fe^3+^ and ROS production in mediating the electrophysiological membrane changes we previously observed during the exposure of *Xenopus* oocytes to crocidolite asbestos. Our findings could represent an important lead for obtaining a better understanding of the relevant processes underlying asbestos toxicity in mammalian cells.

## Results

### Crocidolite-mediated effects on the Xenopus oocyte membrane: the role of H_2_O_2_ and Fe^2+^/Fe^3+^

**Figure 1:**
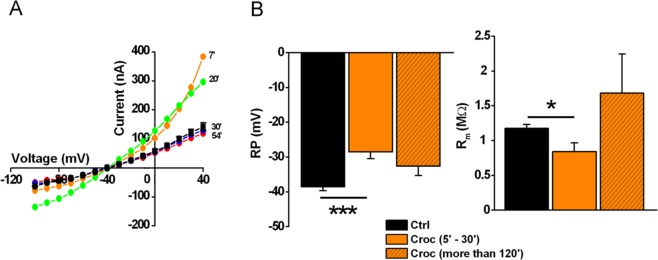
Time-dependency effect of crocidolite (Croc) on the electrical membrane properties of *Xenopus* oocytes. (**A**) *I-V* curve relationships recorded in 4 untreated (Ctrl, black square) and 4 Croc-treated (Croc: 15 μM/ml for 7, 20, 30 and 54 minutes respectively) oocytes. V_h_ = −40 mV, voltage steps: −100 mV to +40 mV, 10 mV intervals. (**B**) Averages of the RP and R_m_ values obtained in Ctrl condition and in cells incubated with Croc at different incubation intervals (5–30 min and more than 120 min). Note at 5–30 min interval there is a significant depolarization of the RP (Ctrl: *n* = 29; Croc: *n* = 37) and a decrease of R_m_ (Ctrl: *n* = 29; Croc: *n* = 37). Oocytes incubated for more than 120 min showed a partial but not significant recovery of the RP (*n* = 9), while the R_m_ was similar to Ctrl cells (*n* = 9). Mean ± SEM. *P < 0.05, ***P < 0.001, One-Way ANOVA (with *Tukey’s post hoc*).

In line with our previous study, crocidolite (Croc) exposure affected the electrical membrane properties of oocytes; specifically, the resting membrane potential (RP) and membrane resistance (R_m_) were significantly reduced with respect to control (Ctrl), while the current amplitudes activated by both negative and positive voltage steps were increased^2^. Fig. 1A shows an example of current-voltage (*I-V*) relationships obtained from non-treated oocytes (Ctrl, *n* = 4) and 4 oocytes incubated in crocidolite respectively for 7, 20, 30 and 54 minutes. In this example, the increase in evoked current amplitude effect started to disappear after 30 minutes of treatment. Despite the variability among different batches of oocytes, the percentage of responsive cells was ~63%. However the response was consistent during the first 30 minutes of incubation while it tended to reverse for incubation times longer than 120 minutes, with the only exception that the RP remained slightly depolarized (Fig. 1B).

These results suggested a time-dependency of the crocidolite-mediated effect on the cell membrane.

**Figure 2:**
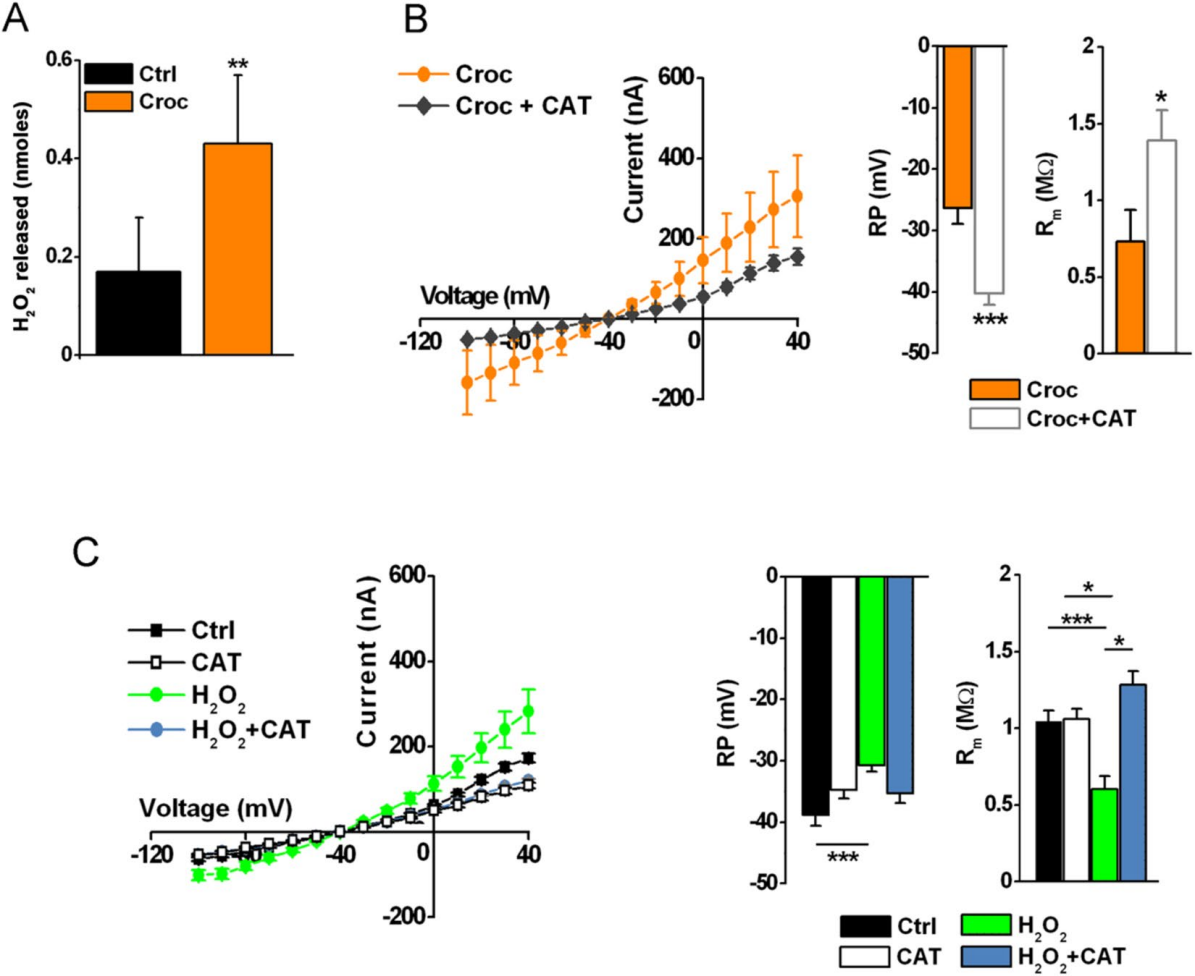
H_2_O_2_ mimics the effect induced by crocidolite. (**A**) Comparison of H_2_O_2_ released by oocytes before and after incubation with Croc (mean ± SD, **P < 0.01, *t-test*, *values are in the text*). (**B**) *left*, *I-V* relationships of Croc-treated cells (15 μg/ml, 5–30 min) without (Croc: *n* = 5) or in the presence of 250 U/ml CAT (Croc + CAT: *n* = 7). V_h_ = −40 mV, voltage steps: −100 mV to + 40 mV, 10 mV intervals. *right*, The RP and R_m_ values of the same oocytes. *P < 0.05, *t-test*, oocytes from same donor. (**C**) *left*, *I-V* relationships of Ctrl (*n* = 20), CAT- (250 U/ml, 5–30 min, *n* = 8), H_2_O_2_− (1 mM, 5–30 min, *n* = 12), and H_2_O_2_ + CAT-treated cells (1 mM H_2_O_2_, 250 U/ml CAT, 5–30 min, *n* = 7). V_h_ = −40 mV, voltage steps: −100 mV to + 40 mV, 10 mV intervals. *right*, Comparison of the RP and R_m_ values of same oocytes. Mean ± SEM. *P < 0.05, **P < 0.01, ***P < 0.001, One-Way Anova test (with *Tukey’s post hoc*). Oocytes from same donors.

One of the mechanisms by which crocidolite fibers are thought to injure cells is by the production of ROS^4^ and there is evidence showing that *Xenopus* oocytes produce ROS endogenously^6^. Here, we planned to measure the production and the extracellular release of hydrogen peroxide (H_2_O_2_) from *Xenopus laevis* oocytes at rest and following asbestos exposure to investigate if ROS could mediate the observed electrophysiological effects of the fibers. We found that untreated cells produced 3.17 ± 1.60 nmoles of H_2_O_2_ in 30 min of incubation (mean ± SD, *n* = 7), while 2.91 ± 1.45 nmoles were found following Croc exposure (mean ± SD, *n* = 7). Of these untreated oocytes, only 0.17 ± 0.11 nmoles were released into the extracellular medium while the exposure to asbestos allowed a release of 0.43 ± 0.14 nmoles of H_2_O_2_ (Fig. 2A), suggesting that crocidolite influenced the *release* of H_2_O_2_ rather than increased the production. Considering that H_2_O_2_ is a key agent in the biological behavior of *Xenopus* oocytes, we decided to evaluate if these cells can adequately dispose of this potentially dangerous molecule. Accordingly, we measured the catalase (CAT) and peroxidase activity of the cells. We found that the former was present in high amount, accounting for 18.6 ± 10.1 units/single oocyte (*n* = 6) of bovine liver catalase taken as standard. We excluded the possibility that asbestos could inhibit catalase activity allowing a higher amount of H_2_O_2_ to injure the cells. Conversely, the activity of this enzyme tended to increase (on the average, not significantly) in the presence of asbestos (15 μg/ml) reaching a value of 25.7 ± 17.9 units/cell (mean ± SD, *n* = 6, ns, *data not shown*). Of note, this activity was found exclusively in the supernatant of cell lysates obtained by cell sonication, suggesting a cytosolic subcellular localization.

The next group of experiments was then aimed at testing whether exogenously - added bovine catalase (CAT) could reverse the effect of crocidolite on the cell membrane properties. Fig. 2B summarizes the results: when cells were pre-incubated in the presence of CAT (250 U/ml, 5 minutes), and then co-treated with crocidolite for 5–30 minutes, the current amplitudes induced by the depolarizing voltage steps were significantly reduced, the RP became more negative (Croc: − 26.2 ± 2.3 mV; Croc + CAT: −40.71 ± 1.69 mV, ***P < 0.001) and the R_m_ increased (Croc: 0.72 ± 0.19 MΩ; Croc + CAT = 1.39 ± 0.18 MΩ, *P < 0.01). In line with these results, the exogenous application of H_2_O_2_ (1 mM) to Ctrl cells, mimicked the effect of Croc, and, as expected, this effect was abolished following the addition of exogenous bovine CAT (250 U/ml), (Fig. 2C).

**Figure 3:**
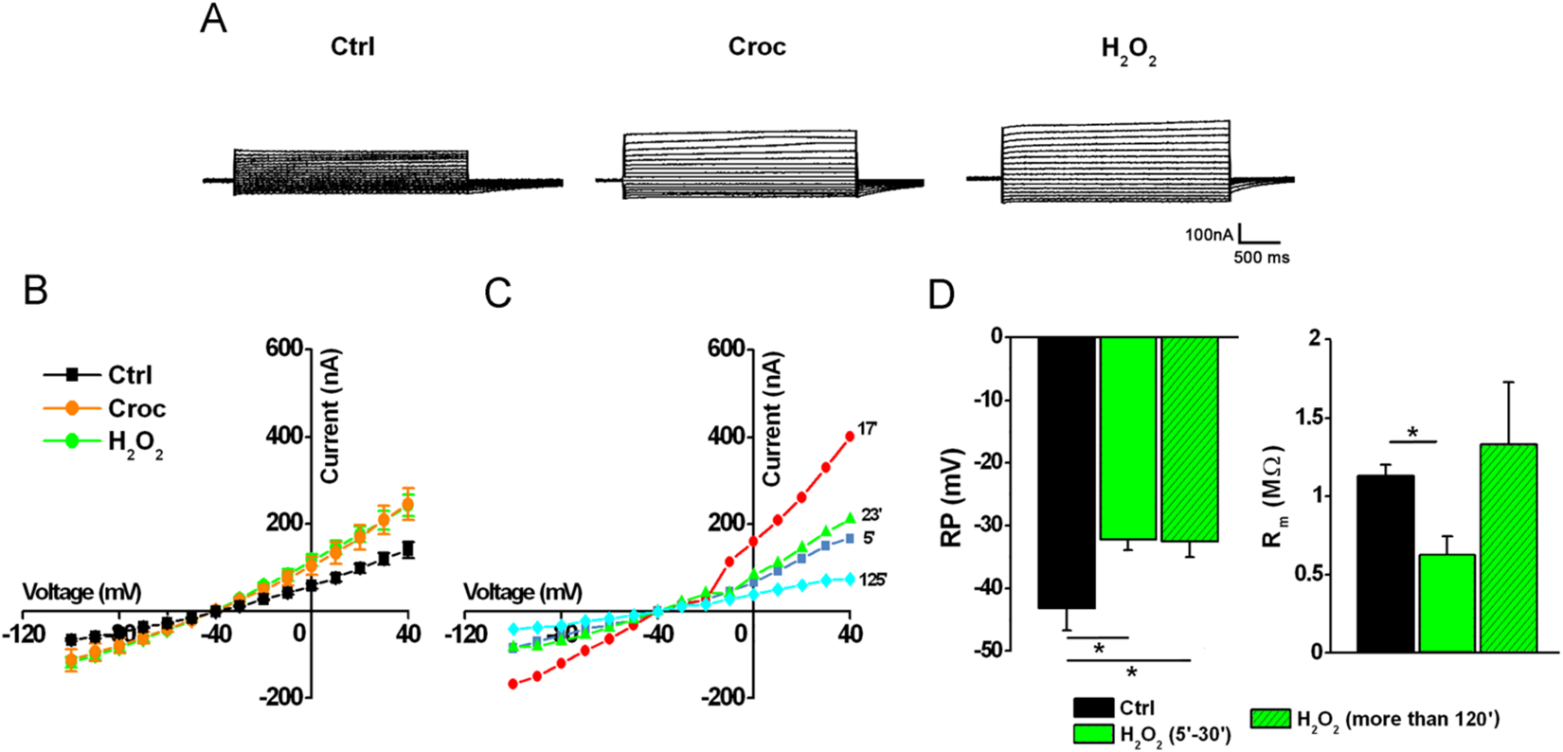
Time-dependency effect of H_2_O_2_ on the oocyte electrical membrane properties. (**A**) Example of currents induced by the voltage step protocol in an untreated oocyte (Ctrl) and in two oocytes treated with Croc (15 μg/ml) or H_2_O_2_ (1 mM). V_h_ = −40 mV, voltage steps: −100 mV to + 40 mV, 10 mV intervals. (**B**) *I-V* relationships of Ctrl cells (*n* = 5), H_2_O_2_ - treated cells (*n* = 5, time interval 5–30 min) and in Croc (*n* = 4, time interval 5–30 min). Oocytes were from the same donor. (**C**) Time course of H_2_O_2_ effect recorded after 5, 17, 23 and 125 min, respectively. (**D**) Effect of H_2_O_2_ on RP and R_m_ after 5–30 min of incubation (*n* = 5) and after more than 120 min (*n* = 3; Ctrl: *n* = 5). *P < 0.05, One-Way Anova test (*Tukey’s post hoc*). Oocytes from same donor.

To further analyze the action of H_2_O_2_ on the cell membrane, another set of experiments was performed and the results are summarized in Fig. 3. In Fig. 3A are shown examples of membrane currents induced by voltage steps in three oocytes, before (Ctrl) and after 20 minutes of incubation with crocidolite (15 μg/ml) or H_2_O_2_ (1 mM). Interestingly, the *I-V* relationships of Croc- treated and and H_2_O_2_-treated cells almost overlapped (Fig. 3B). In line with what was previously observed in Croc-treated cells (see Fig. 1), H_2_O_2_ changed the membrane properties after only a few minutes of treatment, but then its effects disappeared for incubations longer than 120 minutes, with the only exception of the RP, which remained depolarized (Fig. 3C,D).

**Figure 4:**
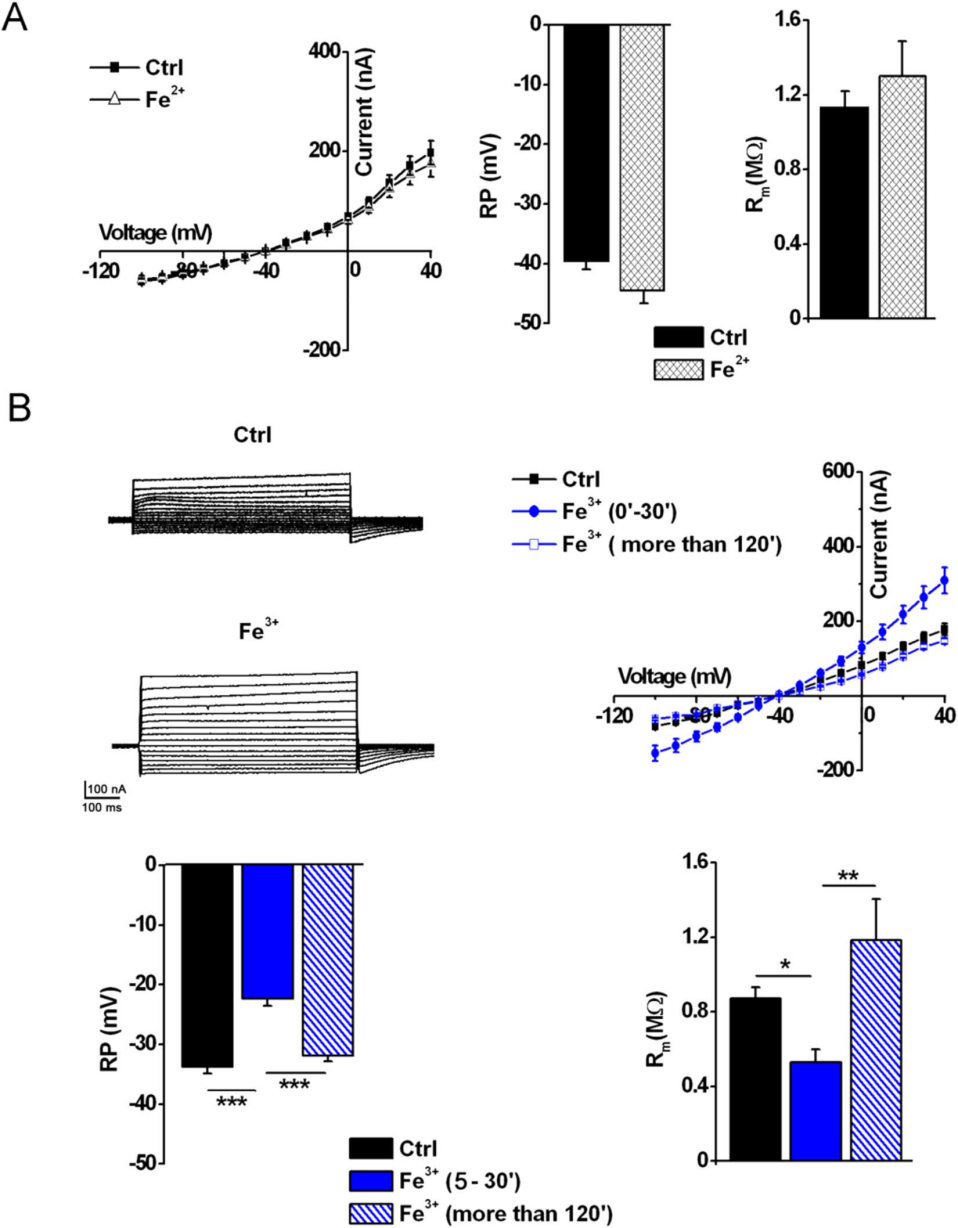
Effect of Fe^2+^ or Fe^3+^ ions on oocyte membrane properties. (**A**) *left*, *I-V* relationships of Ctrl cells and cells treated with Fe^2+^ (1 mM), V_h_ = −40 mV, voltage steps: −100 mV to +40 mV, 10 mV intervals. V_h_ = −40 mV, voltage steps: −100 mV to +40 mV, 10 mV intervals. *right*, The treatment did not change the RP and R_m_ (*n* = 6, *ns*, *t-test*). Both experiments were performed at pH 5. Oocytes from the same donor. (**B**) *left*, Example of recording traces of a Ctrl oocyte and an oocyte after incubation with Fe^3+^ (400 μM). *right*, *I-V* relationships of Ctrl cells (*n* = 8) and cells after a treatment with Fe^3+^ for 5–30 min (*n* = 13) or more than 120 min (*n* = 3). *below*, Comparison of RP and R_m_ values of the same cells (Ctrl: *n* = 13; Fe^3+^ more that 120 min: *n* = 3). Mean ± SEM, *P < 0.05, **P < 0.01, ***P < 0.001, One-Way Anova test (*Tukey’s post hoc*). Oocytes from same donor.

It is also though that the mineral iron content of asbestos fibers is a key factor in inducing cell damage^7^. Therefore, in the following experiments, we tested the possibility that exogenous application of Fe^2+^ or Fe^3+^ might also interact with the oocyte membrane. As summarized in Fig. 4A, when cells were treated with Fe^2+^ up to 1 mM (FeSO_4_, pH 5) the membrane properties remained similar to Ctrl (at pH 5), while in the presence of a lower concentration of Fe^3+^ (FeCl_3,_ 400 μM) the *I-V* curves, and both the RP and R_m_ values were affected in a manner similar (Fig. 4B,C) to that seen after applying crocidolite (15 μg/ml) or H_2_O_2_ (1 mM) (Figs 1 and 3 respectively). All these effects were fully recovered after 120 minutes of incubation. Additional experiments were also performed to evaluate if ferritin, a protein containing a high level of ferric ions, which can express a certain degree of iron-dependent cytotoxicity^8^ may be considered an exogenous source of iron**;** however, ferritin and iron-free apoferritin used as control, did not significantly alter the membrane properties (see Supplementary Fig. S1).

**Figure 5:**
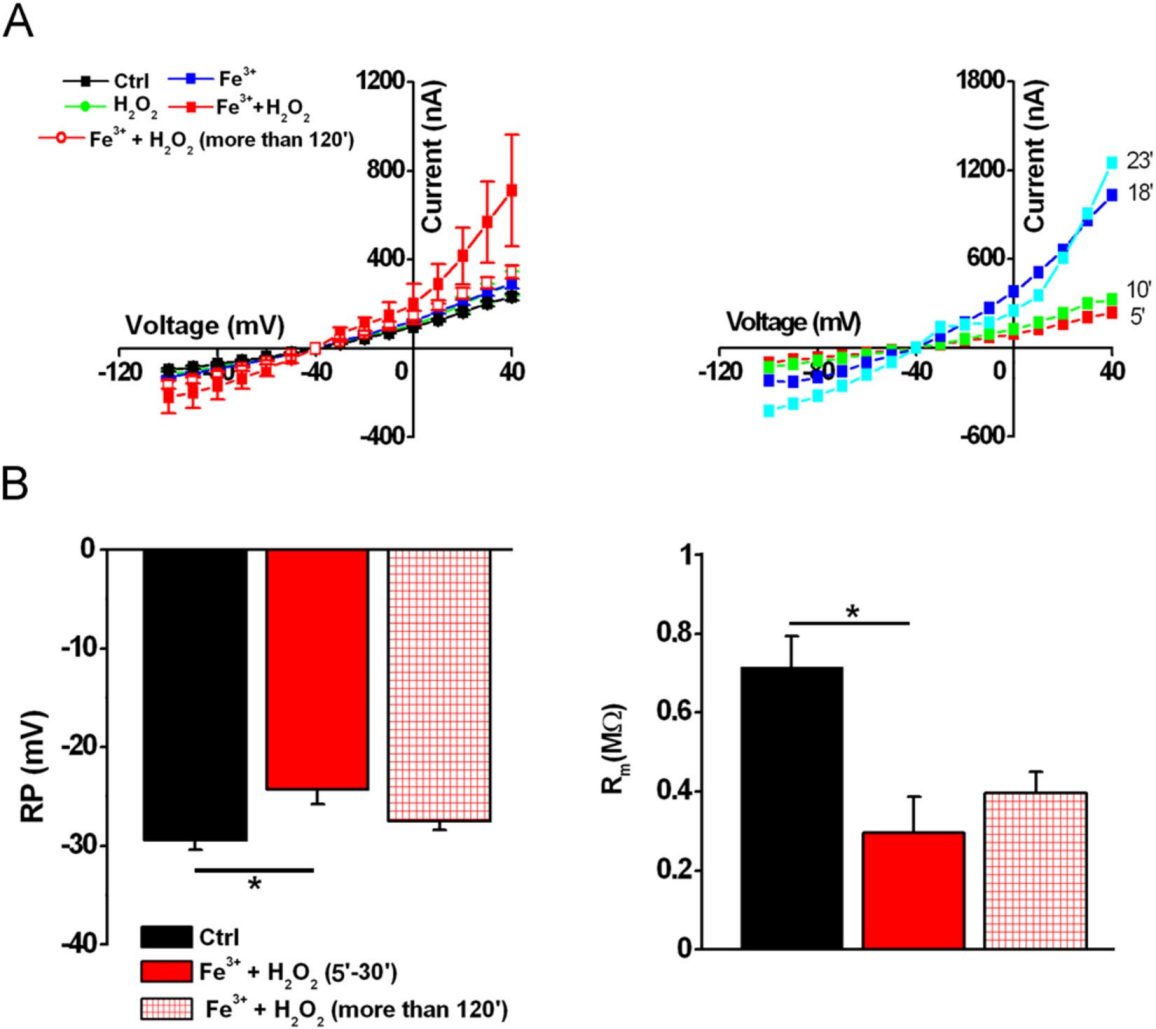
Combined effect of Fe^3+^ and H_2_O_2_ on oocyte membrane properties. (**A**) *left*, *I-V* relationships of Ctrl cell (*n* = 5) and a cell treated with Fe^3+^ (400 μM, *n* = 4, 5–30 min), H_2_O_2_ (1 mM, *n* = 5, 5–30 min) or Fe^3+^ + H_2_O_2_ (400 μM and 50 μM respectively, *n* = 3, 5–30 min). *right*, Comparison of *I-V* relationships obtained in same cells, after the treatment with Fe^3+^ (400 μM) and H_2_O_2_ (50 μM) up to 23 min. V_h_ = −40 mV, voltage steps: −100 mV to + 40 mV, 10 mV intervals. (**B**) The combined treatment significantly depolarized the RP (Ctrl: *n* = 5; Fe^3+^ + H_2_O_2_: *n* = 3) as well the R_m_ (Ctrl: *n* = 5; Fe^3+^ + H_2_O_2_: *n* = 3), both values were partially recovered after a prolonged treatment (*n* = 3). Mean ± SEM. *P < 0.05 One-Way Anova test (*Tukey’s post hoc*). Oocytes from same donor.

**Figure 6:**
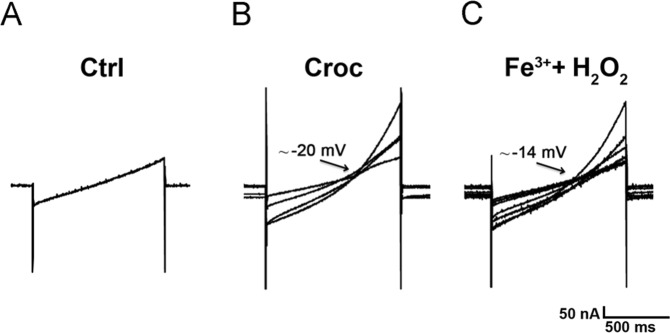
Comparison of currents activated by a voltage-ramp protocol. Example of currents activated by a ramp voltage protocol (from −120 mV to +40 mV, 1 s, V_h_ = −40 V) in a Ctrl cell (**A**) and in oocytes treated with crocidolite (15 μg/ml, 5–30 min, *n* = 4, **B**) or Fe^3+^ + H_2_O_2_ (400 μM and 50 μM, 5–30 min, *n* = 5, **C**). The arrows indicate the mean intersection points with the Ctrl *I-V* (Croc: −20.64 ± 2.5 mV; Fe^3+^ + H_2_O_2_: −14.45 ± 4.76 mV).

Accordingly, when cells were treated with a combination of Fe^3+^ (FeCl_3,_ 400 μM) and H_2_O_2_ (50 μM) the membrane effects were further potentiated (Fig. 5). The time course was similar to that previously observed in the presence of crocidolite, Fe^3+^ and H_2_O_2_ alone, suggesting a common mechanism. The extent of the improvement exceeded the sum of the effects elicited by either compound, suggesting a synergic effect, which was only partially recovered, as shown in Fig. 5B, when the currents were recorded after 2 hours of treatment. In Fig. 6 are shown examples of membrane currents induced by a linear voltage-ramp protocol (−120 to +40 mV; 1 s) in a Ctrl cell (A), and in oocytes treated with Croc (B) or Fe^3+^ +H_2_O_2_ (C). The arrows indicate the average of the *I-V* intersection potentials (indicating the reversal potential of the ionic conductance induced by both treatments) recorded in the treated cells of the two examples. These mean values were not significantly different (Croc: −19.39 ± 1.65 mV, range −2 mV to −29.67 mV, *n* = 19; Fe^3+^ + H_2_O_2_ = −14.45 ± 4.76 mV, range: −6.5 mV to − 29.67 mV, *n* = 5) suggesting a common induced ionic conductance increase.

### The role of cytoskeletal changes following crocidolite exposure

Having shown that similar electrophysiological changes could be induced in the oocytes either by crocidolite, H_2_O_2_ or Fe^3+^, we decided to investigate the nature of the underlying mechanism in terms of the possible morphological changes occurring in the cell surface membrane. Mature oocytes are unable to ingest particulate matter in the brief time employed in our experiments and, as previously suggested, the asbestos fibers can penetrate the cell membrane through “passively” induced lesions^2^. Such lesions of the cell membrane could be repaired rapidly by peripheral actin contraction^9^. To explore this possibility further, we impaired the membrane actin-mediated repair system through treatment with the actin polymerization inhibitor cytochalsin D (CyTD), and investigated if a possible persistence of the lesions induced after CyTD treatment would further influence the membrane properties of the Croc-treated oocytes. We monitored these possibilities by morphological and electrophysiological means.

**Figure 7:**
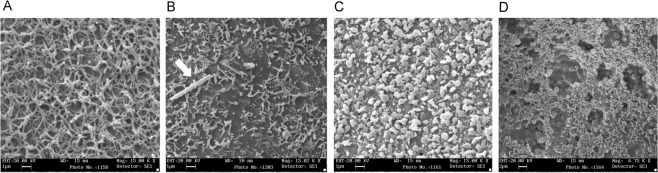
Ultrastructural appearance of the plasma membrane of *Xenopus* oocytes under SEM, taken in those regions that following processing, revealed fractures of the vitelline membrane and showed underneath the surface of the oocyte plasma membrane. (**A**) Shows the plasma membrane appearance of an untreated oocyte and (**B**) following the exposure to asbestos fibers (arrow). The surface appearance was completely changed when the oocyte was treated with cytochalasin D (CyTD, **C**). In (**D**) a CyTD-treated oocyte exposed to [*Croc exposure (60* *min); CyTD alone (60* *min); CyTD* + *Croc co-incubation (60* *min)*].

Figure 7(A–D) shows the surface appearance of oocytes under SEM, either in Ctrl solution (A), in the presence of crocidolite alone (15 μg/ml, B), in the presence of CyTD (5 μM, C) or 5 min preceding the addition of crocidolite (D). The sites of the oocyte surface where the vitelline envelope was detached from the cell membrane (the microvilli of the plasma membrane) were well evident in untreated cells (Fig. 7A). A regular pattern of microvilli was seen as an organized tangled web, where the microvilli delimited some space with the appearance of polygonal structures; no secreted granules were seen. Following the exposure to asbestos fibers, these sites showed a more disordered ultrastructure, with some areas devoid of microvilli and some granules appeared to be secreted (Fig. 7B). The presence of CytD induced an almost complete loss of microvillar structures, the microvilli rounded up and were arranged without a precise scheme (Fig. 7C), whereas in Fig. 7D, the appearance of oocytes exposed to crocidolite in the presence of CytD showed that many “pore-like” lesions were induced, with a mean diameter of 5.0 ± 1.4 μM (SD). The lesions were frequently observed among the inflated microvilli, making evident the underlying secretory granules, many of which were undergoing the secretory process. Interestingly, no asbestos fibers were shown to be associated with these lesions, suggesting that they formed as an indirect effect to the fiber exposure.

**Figure 8:**
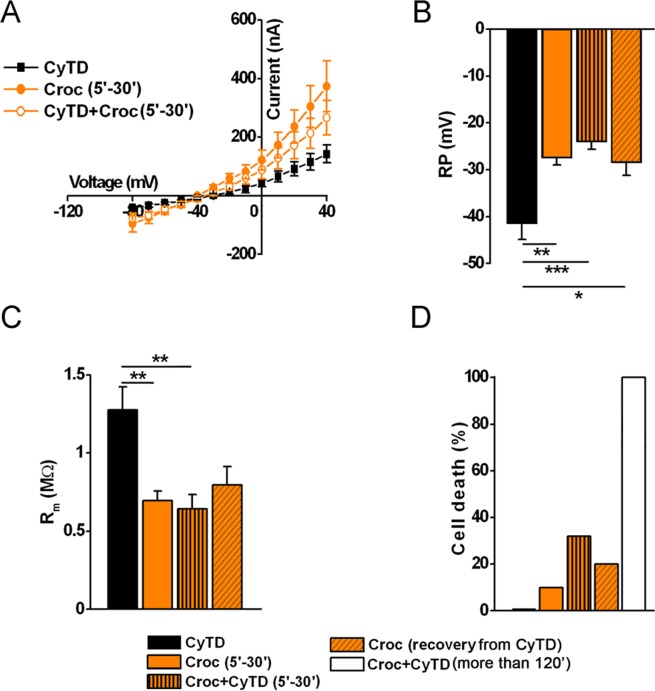
Reversible effect of crocidolite on the oocyte cell membrane was prevented by the presence of cytochalsin D (CyTD). (**A**) *I-V* curve relationships of oocytes treated with CyTD (CyTD: 5 μM, *n* = 8), Croc (Croc: 15 μg/ml, *n* = 41) or CyTD + Croc (CyTD + Croc, *n* = 36). V_h_ = −40 mV, voltage steps: −80 mV to + 40 mV, 10 mV intervals. (**B**) Comparison of RP and (**C**) R_m_ values of the same oocytes and those left in Croc after a 30 min of co-treatment with Croc and CyTD (Croc recovery from CyTD). (**D**) Co-treatment for more than 120 min killed 100% of the cells (CyTD 0%, Croc 10%, Croc + CyTD 32%). Mean ± SEM. *P < 0.05, **P < 0.01, ***P < 0.001, One-Way Anova test (with *Tukey post hoc*). Oocytes from same donors.

These findings suggested that in the presence of CytD, the lesions induced by crocidolite exposure at the membrane level persisted longer than in the absence of CytD. To further confirm these observations, additional experiments were performed to characterize the electrical membrane properties in the presence of the mycotoxin. We first tested the effect of CyTD in control cells. As reported by others^10^, we found that CyTD (5 μM, 30–120 minutes of incubation) did not alter the cell membrane properties (Ctrl: −34.08 ± 1.24 mV, R_m_ = 1.67 ± 0.09 MΩ, *n* = 33; CyTD: RP = −36.94 ± 2.24 mV, R_m_ = 1.96 ± 0.25 MΩ, *n* = 18). Accordingly, when cells were pre-incubated with CyTD and then co-exposed to crocidolite for a short interval (5–30 minutes), the effect observed was similar to that seen in the presence of asbestos alone (Fig. 8A–C). In the former case, however, 32% of the cells displayed a dramatic depolarization of the RP (lower than −10 mV), followed by a consistent reduction of the R_m_ that prevented the voltage clamping of the cell membrane. By removing the CyTD immediately after the short co-treatment (thus leaving the cells in the presence of crocidolite alone) the percentage of dead cells became 20%, while it increased to 100% if the co-treatment lasted more than 120 min (Fig. 8D). While the CyTD effect was partially reversible after brief incubation, after longer incubation the lesions induced by crocidolite became permanent.

**Figure 9:**
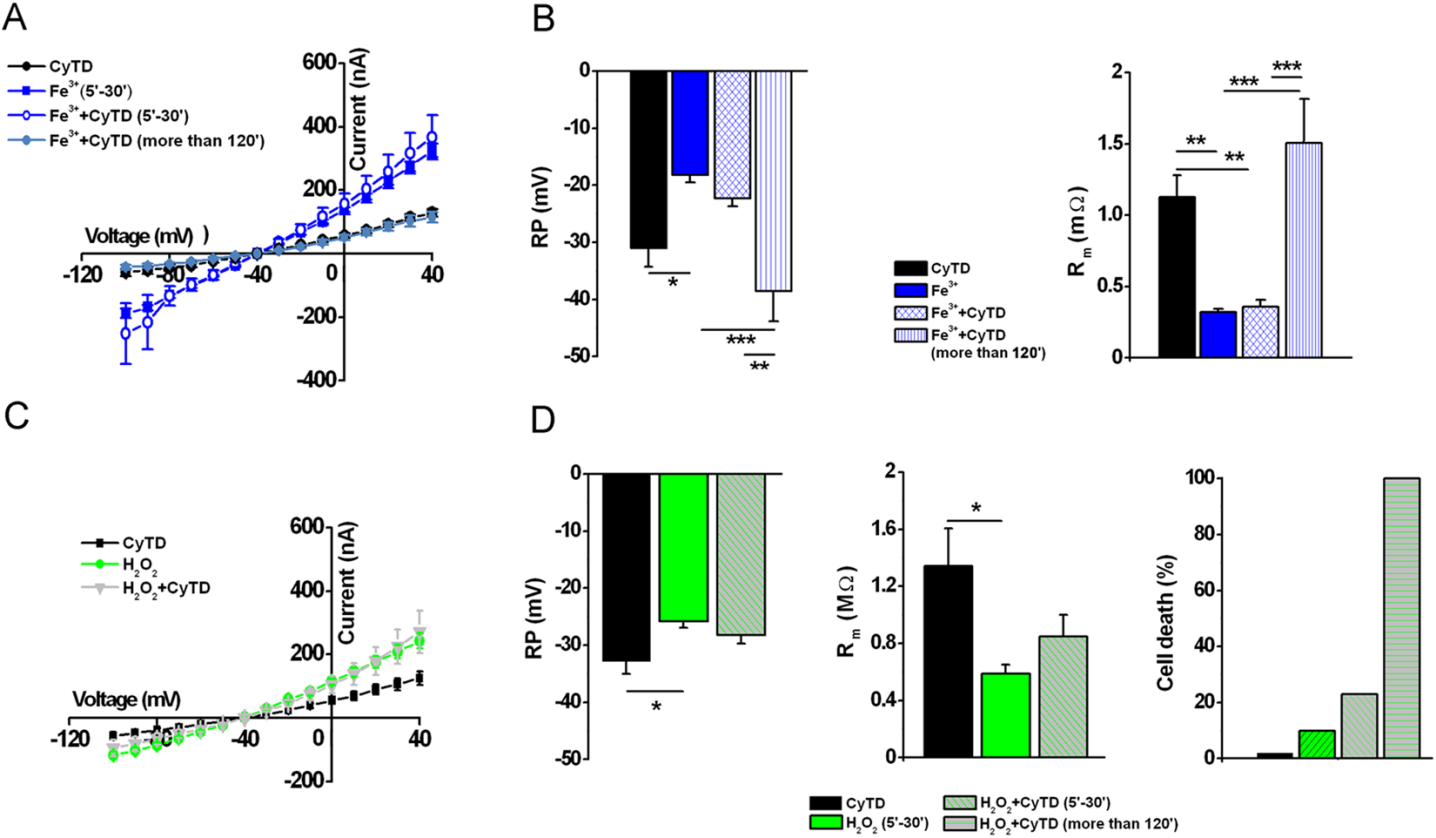
Effect of Fe^3+^ or H_2_O_2_ on the oocyte cell membrane treated in the presence of cytochalsin D. (**A**) *I-V* curve relationships of oocytes treated with cytochalsin D (CyTD: 5 μM, *n* = 5), Fe^3+^ (400 μM, *n* = 5) or Fe^3+^ + CyTD (5–30 min, *n* = 8; more than 120 min, *n* = 4). V_h_ = −40 mV, voltage steps: −100 mV to + 40 mV, 10 mV intervals. (**B**) Comparison of RP and R_m_ values of the same oocytes. Mean ± SEM, *P < 0.05, **P < 0.01, ***P < 0.001, One-Way Anova test (*Tukey post hoc*). Oocytes from same donor. (**C**) *I-V* curve relationships of oocytes treated with cytochalsin D (CyTD: 5 μM, *n* = 5), H_2_O_2_ (1 μM, *n* = 5), H_2_O_2_ (1 mM) + CyTD (5 μM, 5–30 min, *n* = 5). V_h_ = −40 mV, voltage steps: −100 mV to +40 mV, 10 mV intervals. (**D**) Comparison of RP and R_m_ values of the same oocytes. Note that long incubation with H_2_O_2_ + CyTD killed 100% of the cells. Mean ± SEM *P < 0.05, One-Way Anova test (*Tukey post hoc*). Oocytes from same donor.

The last question we tried to answer was the role of H_2_O_2_ and Fe^3+^ in the context of the “pore-like” lesions. To address this, we set up a similar experiment as described above; cells were co-treated with CyTD (5 μM) +H_2_O_2_ (1 mM) or CyTD (5 μM) +Fe^3+^ (400 μM) at different time intervals (5–30 minutes, and more than 120 minutes). As before, the results were compared to those obtained from oocytes treated with CyTD alone, coming from the same donor (Fig. 9). Surprisingly, only CyTD-treated cells incubated in the presence of H_2_O_2_ died after incubation longer than 120 minutes (Fig. 9D), while in the presence of either H_2_O_2_ alone or Fe^3+^ + CyTD the effect remained reversible. Similarly to the effect of crocidolite, the co-treatment in the presence of Fe^3+^ (400 μM) + H_2_O_2_ (100 μM) and CyTD (5 μM) killed about 70% of cells after 2 hr, but 25% of the cells when the H_2_O_2_ concentration was 50 μM (*data not shown*).

### Crocidolite may mediate its activity by modulating calcium-activated chloride channels

**Figure 10:**
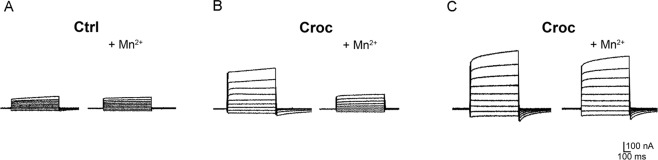
Crodidolite-mediated effect may involve Ca^2+^-activated Cl^−^ channels. Effect of Mn^2+^ (5 mM) on a non-treated (**A**) and in two crocidolite-treated oocytes (oocytes form the same donor, **B**,**C**). V_h_ = −70 mV (voltage steps: −100 mV to +60 mV, 20 mV intervals). In (**B**) Mn^2+^ produces a substantial reduction of the Croc-induced outward currents (on average the currents at +20 mV decreased from 324.63 ± 96.63 nA to 189.22 ± 27.68 nA, mean ± SD, *n* = 4, *P < 0.05) whereas in (**C**), it has a minimal effect, suggesting that the effects of Croc may involve multiple conductance mechanisms in different cells.

One of the possible mechanisms through which crocidolite could affect the cell membrane conductance is by modulating the activity of endogenous ion channels. It is well known that *Xenopus* oocytes express different types of endogenous channels^11^, including a Ca^2+^-activated Cl^−^ channel (TMEM16A)^12^. Here we found than in 4 out of 5 treated cells, the Croc-induced currents became significantly reduced in the presence of Mn^2+^ (5 mM, see example of Fig. 10B), which is known to block the endogenous Ca^2+^-activated Cl^−^ channel^13^. However, in one treated oocyte, the effect was not so evident (shown in Fig. 10C), suggesting that asbestos may also affect another conductance, “insensitive” to Mn^2+^, in the oocyte cell membrane.

## Discussion

Considerable evidence shows that asbestos can affect biological cells by a direct cell membrane interaction^14–16^. Recently, we demonstrated that *Xenopus* oocytes represent a suitable model for studying these kinds of interactions^2^. We found that exposure to crocidolite affected ~63% of the cells tested by inducing an increase of the outward currents activated by voltage clamp steps, as well as modifying the resting membrane potential and membrane resistance; these effects were time-dependent and partially reversible. Consequently, the percentage of responsive cells excludes cells in which the effect had already vanished. Moreover, we found that H_2_O_2_ production and exposure/access to Fe^3+^ ions were most likely the main candidates responsible for inducing such effects.

A number of physicochemical properties are responsible for inducing asbestos-correlated disease^17–19^, including iron content and ROS production^20^. Crocidolite generates the highest amount of ROS compared to other asbestos fibers, and the production is correlated with the mobilizable surface iron found in these types of fibers, which can catalyze the formation of hydroxyl radicals by either the Fenton or Haber-Weiss reactions^4,20^. Therefore, in this study, we focused on the possible role of H_2_O_2_ and accessible Fe^2+^/Fe^3+^ ions in the crocidolite-mediated effects.

Like most cells, *Xenopus* oocytes produce H_2_O_2_ endogenously^6,21^, which is normally detoxified by catalase, present in large amounts in these cells. As a result, only very small amounts reach the extracellular environment, despite the high membrane permeability to H_2_O_2_^22,23^. In the presence of asbestos fibers, we envisage that the additional H_2_O_2_ produced by the oocytes is diverted from the catalase-mediated detoxification, and is thus released through the plasma membrane in higher amounts, since no barrier is posed to H_2_O_2_ diffusion; the same amount of that released is active inside the cell. Accordingly, it can modify its electrical membrane properties. It may be noted that asbestos fiber exposure, at the concentrations employed here, did not inhibit catalase; rather, it *increased* its enzymatic activity (not shown) as it does for the activity of peroxidases and chymase^24^. Thus, H_2_O_2_ may contribute to the membrane-injuring effect of crocidolite. Interestingly, applied H_2_O_2_ reproduced the effect of asbestos, and CyTD in combination, hindered the cell recovery as it did in asbestos-treated oocytes.

We previously showed that iron chelators prevented the crocidolite effects, suggesting a possible involvement of iron in the underlying mechanism of action^2^. In oocytes, iron is localized at the animal pole, where it is involved in the interaction with enzymes, transcription factors and redox sensor^25^. It is usually sequestered by ferritin^26,27^ in order to prevent the accumulation of the free ions. These are indeed responsible for cell damage induced by the Fenton reaction, which promotes the production of ROS^15,17^. Crocidolite fibers contain high amounts of iron (~27% w/w (both Fe^2+^ and Fe^3+^); formula: [(Na_2_(Mg,Fe)_6_Si_8_O_22_(OH)_2_]), and here we found (as expected, since Fe^2+^ cannot enter *Xenopus* oocytes^28^), that only applied Fe^3+^ was able to affect the electrical membrane properties in an asbestos-like manner. Thus, the exposure to crocidolite, H_2_O_2_ or Fe^3+^, although to different extents, produced the same type of electrophysiological changes. Interestingly, in all these cases, the initial changes in electrical membrane properties were almost recovered after ~120 min of incubation.

We also report here, further evidence supporting the “pore hypothesis” for the damaging actions of asbestos^24,29^. We showed that the actin cytoskeleton, which is involved in the process of repairing membrane lesions in *Xenopus* oocytes^30^, modulates the cell response to asbestos, preventing the lesion from becoming permanent as suggested by SEM pictures and electrophysiological evidence.

ROS affects other electrophysiological membrane parameters in many cell types including: variations of membrane current and potential, ionic gradients, and loss of excitability^31^. Membrane depolarization is one of the earliest membrane modifications, followed by an increase in leak currents that affect the membrane resistance^31,32^. H_2_O_2_, for instance, can mediate its effect by acting on different ion transporters/channels, but also by inducing alterations in cell membrane fluidity and “leakiness”. This kind of injury, associated with membrane lipid peroxidation, is reversible. In *Xenopus* oocytes, it is still unclear how H_2_O_2_ interacts with the membrane; some authors have suggested the activation of an endogenous non-selective cationic conductance responsible for the membrane depolarization^33,34^, while others observed the activation of chloride currents^35,36^. In the presence of crocidolite, and Fe^3+^ + H_2_O_2_, we found that the voltage ramp protocol activated currents that intersected with the control *I-V* at a range close to the chloride equilibrium potential (E_Cl_) in *Xenopus* oocytes (≈ − 22 mV)^37^. Recently, it has been shown that an endogenously expressed Ca^−2+^-activated Cl^−^ channel (TMEM16A)^12,38^, selectively blocked by Mn^2+^ ^12^, can be modulated by ROS similarly to what we observed in crocidolite-treated cells^39^. Although highly suggestive, at present, we cannot exclude the involvement of other conductances in the oocyte cell membrane. These aspects therefore deserve to be further investigated.

The three experimental conditions we employed allowed us to precisely dissect the likely molecular mechanism of action of crocidolite on the oocyte cell membrane. Firstly, the membrane modification induced by crocidolite was inhibited by iron chelators^2^ and was catalase sensitive, thus its action was iron and H_2_O_2_-dependent. Accordingly, iron-free multiwall carbon nanotubes (MWCNTs) or functionalized with pluronic acid: f-MWCNTs), which do not contain iron, are completely ineffective on this preparation (see Supplementary Fig. S2). Secondly, exogenously-added H_2_O_2_ induced the same modifications elicited by asbestos and the lesions induced (obviously catalase sensitive), also became permanent following the inhibition of actin repair mechanisms with CyTD. Thirdly, exogenously-added Fe^3+^ also elicited the same membrane modification induced by asbestos, although to a weaker extent. Furthermore, also in this case, the effect appeared to be catalase sensitive (see Supplementary Fig. S3). Lastly, the combined action of H_2_O_2_ and Fe^3+^ ions triggered a synergistic effect, identical or even stronger than that elicited by asbestos fibers, which in the presence of CyTD, caused the death of the oocytes, suggesting the trigger of a self-increasing chain reaction. The effect of CyTD likely depends on the persistence of membrane lesions (pore-like), which cannot be repaired due to the cytoskeleton disruption. Since the lesions appear to be larger than the average asbestos fiber width, we cannot exclude that the effect of CyTD could be explained also by the entry of more fibers through the pores, together with alteration of ion fluxes.

These findings make a link between the effects induced by the different experimental conditions employed, and suggest that the responses are very likely dependent on ROS production.

**Figure 11:**
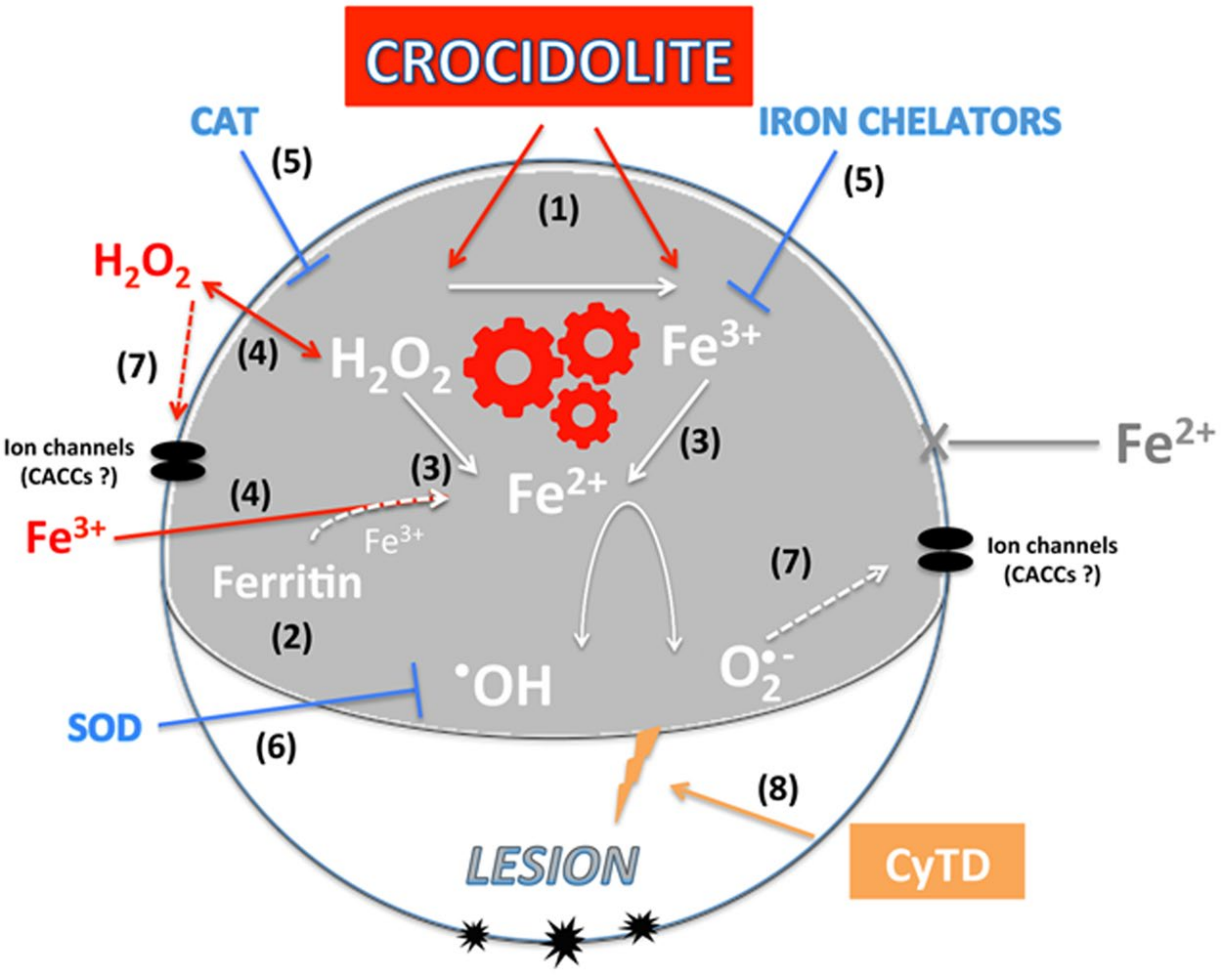
Schematic summary of the sequence of common mechanisms which either crocidolite or H_2_O_2_ + Fe^3+^ may share for inducing electrophysiological changes and cell damage in *Xenopus* oocytes: (**1**) Exposure to crocidolite fibers allows more H_2_O_2_ to become available, which in turn induces Fe^3+^ to be released from the fibers themselves (**2**). Free Fe^3+^ also derives from ferritin in the presence of H_2_O_2_ (**3**). Fe^3+^ can be reduced to Fe^2+^ by H_2_O_2_. Fe^2+^ in turn, triggers ROS production by reacting with H_2_O_2_ (**4**). The same reaction can be triggered by the exogenous addition of either H_2_O_2_, which can react with ferritin-released Fe^3+^, or Fe^3+^ that reacts with the small amount of H_2_O_2_ produced by resting *Xenopus* oocytes (**5**). As expected, iron chelators or catalase (CAT) prevent the changes induced by either crocidolite or H_2_O_2_ + Fe^3+^ (**6**). The main character for inducing membrane lesion is most likely OH°, as superoxide dismutase (SOD), which allows O_2_^−^ dismutation, failed to inhibit these changes. The final membrane effect/lesion may be twofold:(**7**) a modification of the function of an endogenous chloride channel (possibly a calcium-activated chloride channel CACC) and (**8**) the formation of membrane “pores”, revealed morphologically when the cortical actin repair mechanism is inactivated by CyTD.

On this basis, we suggest that asbestos fiber exposure induces membrane lesions, which become permanent when the healing mechanisms are hindered and that these lesions are induced by the combined action of Fe^3+^ ions and H_2_O_2_. We hypothesize that the lesions induced by asbestos fibers are triggered by a chain reaction supported by the continuous provision of both H_2_O_2_ and Fe^2+^ ions. We believe that in asbestos-exposed *Xenopus* oocytes, the iron source may be provided by the fibers themselves^40^ and/or ferritin^41^, present in oocytes:^27^ both fibers and endogenous ferritin (the exogenously-added being inactive: as shown by dedicated experiments see Supplementary Fig. S1) can release iron ions in the presence of H_2_O_2_ (ferrous and ferric from fibers and ferric from ferritin^41^). The source of H_2_O_2_ is in this case, is the amount that escapes the catalase-induced dismutation. The finding that asbestos seems to allow more H_2_O_2_ to escape from detoxification mechanisms could be the consequence of either/both a modification of the H_2_O_2_ clearance mechanisms and/or of the microenvironment where the H_2_O_2_-producing enzymes are working. We did not pursue this aspect further, which however deserves to be investigated, both in *Xenopus* oocytes and in human lung cells, considering that H_2_O_2_ availability is a key factor in asbestos- induced injury. The ferrous iron can interact with H_2_O_2_ and trigger the Fenton reaction. The finding that extracellular H_2_O_2_ produces the same effect in oocytes as adding asbestos is completely in agreement with this hypothesis. H_2_O_2_ can diffuse passively through the membrane^22,23^, trigger the release of iron from ferritin^41^, reduce Fe^3+^ to Fe^2+^ ^42^ and finally trigger the Fenton reaction^15,43^. The addition of exogenous Fe^3+^, as expected, gave a weak effect. It can be taken up by *Xenopus* oocytes, and could be reduced inside to Fe^2+^ (impermeant^28^ by the small amount of H_2_O_2,_ which in resting *Xenopus* oocytes, can escape from the catalase dismutation. The electrophysiological effect of adding Fe^3+^ was completely reversible, also in the presence of CyTD, suggesting that no significant membrane lesion was produced. However, following the addition of even a low amount (50 μM) of H_2_O_2_, a synergistic reaction was triggered which induced also a significant proportion of oocyte deaths, due to permanent membrane lesions (Fig. 11). The chain reaction starts with Fe^2+^, which in the presence of H_2_O_2_, triggers production of OH^•^ radicals and Fe^3+^ (Fenton reaction; 15, 43). We suggest that the cytotoxic species, capable of inducing the “pore-like” lesions, may be OH^•^ rather than ^•^O_2_−, since the latter should be rapidly dismutated by superoxide dismutase (SOD), (abundant in *Xenopus* oocytes^44^) and cannot restore its membrane properties in Croc-exposed *Xenopus* oocytes (see Supplementary Fig. S4) to produce even more H_2_O_2_ for supplying the chain reaction. We think that this reaction will last until the sufficient amount of both H_2_O_2_ and ferrous iron will be supplied, and vanish thereafter. During its activity, OH^•^ will be produced and cause membrane damage, responsible for the surface modification observed under SEM. This damage became permanent and caused cell death when the actin repair mechanisms were inhibited. Otherwise, the repair mechanisms allowed the complete recovery of membrane properties at the end of the chain reaction activity.

In our model, the asbestos fibres may be one source of iron. It is very likely that the fibres interacting with the plasmamembrane or those which have reached the cell interior may be more important, since they are expected to come in contact with the higher H_2_O_2_ concentration. However, we cannot exclude a partial contribution also of fibers remaining on the outside.

H_2_O_2_ seems to be provided by the cell itself and by the superoxide (coming from the reduction of molecular oxygen by Fe^2+^) dismutation. In other cell types, the source of iron and H_2_O_2_ could be different. For example, inflammatory cells^14^ produce ROS, following exposure to fibers and take up Fe^+2^ for subsequent storage in ferritin in the oxidized form. Interestingly, in asbestos-exposed cells, ferritin is newly synthetized^45,46^ and iron is taken up by the cells and may even supplement the crocidolite iron content^47^, which can compensate for that released by H_2_O_2_. So, in these cells, the production of H_2_O_2_ and of Fe^2+^ could be maintained for a long time causing a continuous production of OH^•^.

Our paper however, d**e**scribes one of the mechanisms, perhaps the main one, triggered by crocidolite, where the availability of fiber surface iron^15^ and the production of ROS can trigger a chain reaction capable of injuring the cell membrane, initiating an inflammatory reaction and inducing cell death. We also suggest that all the described changes would follow the passive entry of fibers inside the cells, which can contribute significantly in determining the membrane lesion and the “pore formation”. Further studies are necessary to investigate if the same reaction can also damage nucleic acids and start neoplastic transformation.

In conclusion, *Xenopus* oocytes allowed us to focus on a specific effect of crocidolite, which deserves to be tested also on human lung cell lines. Much evidence suggests that asbestos fibers damage cells through ROS production^14,15^. Our data confirm that the fibers, after reaching the cell interior, can trigger a ROS-mediated damaging effect capable of acting until iron and H_2_O_2_ are provided. In our model, the damage could be repaired by the contribution of the cytoskeleton.

## Materials and Methods

### Asbestos fiber suspensions

An analytical Standard UICC (reference batch: South African 12001–28–402704-AB) sample of crocidolite was obtained from SPI-CHEM, West Chester, Pennsylvania, re-suspended in PBS at a final concentration of 10 mg/ml, and stored at 4 °C until use. The fiber size parameters of the asbestos UICC standard have been described in detail by Kohyama *et al*.^48^. Our standard fibers spanned from 0.5 (accounted for 10%) to 20–100 μM (accounted for about 5%) in length and from 0.1 (accounted for about 5%) to 0.8–1.0 μM (accounted for about 5%) in width. On average, the fibers had a length of 2.5 ± 2.0 (SD) μM and a width of 0.33 ± 2.1 (SD) μM. So the smaller fibers had a 0.1 (width) and a length of 0.5 μM. At the concentration, continuous mixing and temperature used in our experiments, no fiber aggregation occurred as judged by optical microscope analysis.

### Oocyte preparation

Animal care and treatment were conducted in conformity with institutional guidelines in compliance with national (Italian Ministry of Health, authorization number 717/2015, released on July 17, 2015) and international laws and policies (European Economic Community, Council Directive 63/2010 Italian D.L. 26/2014). Adult female *Xenopus laevis* frogs were fully anesthetized in cold 0.17% MS-222 solution. Ovaries were isolated and the layer of follicular cells mechanically removed. Oocytes were treated with 0.5 mg/ml collagenase (35 min) and maintained in Barth’s medium (NaCl 88 mM, KCl 1 mM, Ca(NO_3_)_2_ 0.33 mM, CaCl_2_ 0.41 mM, MgSO_4_ 0.8 mM, NaHCO_3_ 2.4 mM, HEPES 10 mM, adjusted to pH 7.4 with NaOH), with addition of gentamicin (50 µg/ml).

### Electrophysiological recordings

Electrophysiological recordings were performed 24 h after the isolation to allow healing of the oocyte membrane from damage caused by the collagenase. Ten-fifteen oocytes (stage VI) in a 1.5-ml Eppendorf tube were incubated in 1 ml of Ringer’s solution (NaCl 115 mM, KCl 2 mM, CaCl2 1.8 mM, HEPES 5 mM, adjusted to pH 7.4 with NaOH) without (Ctrl cells) or in test conditions (15 µg/ml of crocidolite fibers, 400 μM FeCl_3_ at pH 7.4, 1 mM FeSO_4_ at pH 5, H_2_O_2_, 250 U/ml catalase or cytochalasin D 5 μM), in both cases, under continuous mixing accordingly with the different experimental conditions (wheel, 7 revolutions/min). The concentration of H_2_O_2_ added varied from 50 μM to 1 mM for obtaining a significant effect. Hydrogen peroxide in the absence of oocytes did not show any decay during all the incubation times considered as judged by the HVA method (see below). This variability is probably due to the variable amount of endogenous catalase that oocytes contain in different experiments (see Results section).

Glass recording microelectrodes were filled with KCl (3 M), with a tip resistance of 0.5–2 MΩ, and connected to an amplifier (Oocyte Clamp OC- 725 C). During the recordings, the cells were continuously superfused with a Ringer’s solution in a purpose-designed recording chamber (RC-3Z, Warner Instruments, Hamden, Connecticut) at room temperature (23 °C). The Ringer’s solution was applied using a constant perfusion system (7 ml/min, VC-8 perfusion system, Warner Instruments), and the flux speed was routinely controlled and maintained constant during each set of experiments^49^. The resting membrane potentials (RPs) of the oocytes were recorded 3–5 min after impalement, when the values were more stable. The membrane input resistances (R_m_) were estimated from the slope of *I*-*V* relationships measured at −100, −90, −80, and −60 mV. The *I-V* curve relationships were obtained by using a protocol: from a holding potential of − 40 mV, the oocytes were clamped from − 100 to +40 mV (3 s), 10 mV intervals. The current amplitudes were measured at the steady state. For the linear voltage ramp protocol oocytes were held at − 40 mV and a linear voltage ramp from − 120 to +40 mV (1 s) was applied. For recording the currents blocked by Mn^2+^, we used a holding potential of −70 mV and then voltage steps from −100 mV to +60 mV (20 mV intervals, 1 s duration). To reduce the variability of oocytes coming from different frog donors, the results were usually compared among oocytes of the same batches^50^. The oocytes were considered “dead” when the resting membrane potential was lower than −10 mV and it was not possible to clamp stably the voltage of the cell membrane.

### Hydrogen peroxide production and release

H_2_O_2_ released was measured fluorimetrically by the homovanillic acid (HVA) method which, being cell impermeable, is able to trap the amount of H_2_O_2_ which escapes from the cell. The procedure is based on the conversion of the non-fluorescent HVA to the highly fluorescent 2,2′ dihydroxy 3,3′ dimethoxy diphenyl 5,5′ diacetic acid, by horseradish peroxidase (HRP) in the presence of H_2_O_2_^51^. Briefly, HVA 0.8 mM and HRP 20 μg/ml were included in 1 ml Ringer solution together with 5 oocytes and, when indicated, asbestos fiber crocidolite 15 μg/ml. The fluorescence developed after 30 min incubation at RT (indicating the H_2_O_2_ released and simultaneously trapped), was read in a Perkin-Elmer spectrophotofluorimeter 650.10 s (λ ex 315 nm, λ em 425 nm). Known amounts of H_2_O_2_ were employed as an internal standard. The total amount of H_2_O_2_ produced,was evaluated by microinjecting 9 nl of the 10 μM CM-H2DCF-DA dissolved in DMSO (INVITROGEN: Molecular Probes), or DMSO into 5 oocytes for each sample. Following 30 min of incubation at RT with or without asbestos fibers, the oocytes were disrupted by sonication, centrifuged at 10,000 × g (Eppendorf microcentrifuge) and the fluorescence developed in the supernatant, read in a Perkin-Elmer spectrophotofluorimeter 650.10 s (λ ex 503 nm, λ em 529 nm). The increment of fluorescence developed by the addition of known amount of H_2_O_2_ to the resting supernatant was taken as internal standard.

### Catalase activity

The activity of Xenopus oocytes and of bovine liver catalase (used as standard; SIGMA) was assayed spectrophotometrically, by following the disappearance of 30 mM H_2_O_2_ absorbance at 230 nm with an Perkin Elmer Lambda 5 recording spectrophotometer as was previously described^52^. Briefly, 10–15 oocyte suspension in 1–2 ml of Ringer’s solution were sonicated (Bandelin Sonopuls sonifier, Bandelin Electronic, D12207 Berlin, Germany) at 50% power for 30 sec) and centrifuged at 10,000 × g at 4 C° for 5 min. Enzyme activity was assayed in the supernatant.

### Scanning electron microscopy (SEM)

The procedure to analyse the oocyte samples by SEM was previously described elsewhere^29^ Control and treated oocytes were fixed with 2.5% glutaraldehyde (Serva, Heidelberg, Germany) in Ringer’s solution at room temperature for 20 min, rinsed in Ringer and post-fixed in 1% osmium tetroxide in PBS for 30 min. Afterwards, samples rinsed in Ringer were dehydrated in ascending ethanol concentrations (35, 50, 70, 90, 100%) and transferred in 100% ethanol to a critical point dryer (Bal-Tec; EM Technology and Application, Furstentum, Liechtenstein) and dried through CO_2_. Coverslips were mounted on aluminum sample stubs and gold coated by sputtering (Edwards S150A apparatus, Edwards High Vacuum, Crawley, West Sussex, UK). SEM images were obtained using a Leica Stereoscan 430i scanning electron microscope (Leica Cambridge Ltd., Cambridge, UK). For each sample observed by SEM, many photomicrographs at different magnifications were stored. SEM, imaging was performed at a range of accelerating voltages of 20 kV, working distance of 17–18 mm and beam currents of 0.08–0.1 nA were used.

### Reagents

MS22 (ethyl 3-aminobenzoate methanesulfonate 98%), collagenase Type I, cytochalasin D (dissolved in DMSO), superoxide dismutase, horseradish peroxidase (HRP, SIGMA), homovanillic acid (HVA), catalase from bovine liver, H_2_O_2_, apoferritin from equine spleen, and ferritin from equine spleen were purchased from SIGMA. Gentamicin Sulfate (50 mg/ml) were from LONZA. Pristine multi-walled carbon nanotubes (MWCNTs) kindly supplied from the laboratory of Prof. Maurizio Prato (Dipartimento di Scienze Chimiche e Farmaceutiche University of Trieste) were re-suspended at 0.5 mg/ml in 0.5% pluronic solution made in distilled water.

### Data analysis

Data acquisition and analyses were performed by WinWCP version 4.1.2 Strathclyde Electrophysiology software, kindly provided by Dr John Dempster (Glasgow, United Kingdom). Prism 3.0 and Origin 7 were used for the statistical analysis. All data passed the normality test. Statistical significance for comparison between different groups was established using a Student’s t test (*t-test*) when comparing two groups, One-Way ANOVA followed by Tukeys’s post hoc test for multiple comparisons. All values are expressed as mean ± SEM or mean ± SD, as indicated in the legends. *P* values < 0.05 were considered as significant.

## Supplementary information

Supplementary info

## References

[1] Donham KJ, Merchant JA, Lassise D, Popendorf WJ, Burmeister LF (1990). Preventing respiratory disease in swine confinement workers: intervention through applied epidemiology, education, and consultation. Am. J. Ind. Med..

[2] Bernareggi A (2015). *Xenopus laevis* oocytes as a model system for studying the interaction between asbestos fibres and cell membranes. Toxicol. Sci..

[3] Xu A, Zhou H, Yu DZ, Hei TK (2002). Mechanisms of the genotoxicity of crocidolite asbestos in mammalian cells: implication from mutation patterns induced by reactive oxygen species. Environ. Health Perspect..

[4] Srivastava RK, Lohani M, Pant AB, Rahman Q (2010). Cyto-genotoxicity of amphibole asbestos fibers in cultured human lung epithelial cell line: role of surface iron. Toxicol. Ind. Health.

[5] Kamp DW, Weitzman SA (1999). The molecular basis of asbestos induced lung injury. Thorax..

[6] Taglialatela M (2007). Regulation of the human ether-a-go-go related gene (HERG) K+channels by reactive oxygen species. PNAS.

[7] Fubini B, Mollo L (1995). Role of iron in the reactivity of mineral fibers. Toxicol Lett..

[8] Krenn MA (2015). Ferritin-stimulated lipid peroxidation, lysosomal leak, and macroautophagy promote lysosomal “metastability” in primary hepatocytes determining *in vitro* cell survival. Free Radic Biol Med..

[9] Ryabova LV, Vassetzky SG (1997). A two-component cytoskeletal system of *Xenopus laevis* egg cortex: concept of its contractility. Int. J. Dev. Bio..

[10] Peter AB, Schittny JC, Niggli V, Reuter H, Sigel E (1991). The polarized distribution of poly(A+)-mRNA-induced functional ion channels in the *Xenopus* oocyte plasma membrane is prevented by anticytoskeletal drugs. J. Cell. Biol..

[11] Terhag J, Cavara NA, Hollmann M (2010). Cave canalem: how endogenous ion channels may interfere with heterologous expression in *Xenopus* oocytes. Methods.

[12] Schroeder BC, Cheng T, Jan YN, Jan LY (2008). Expression cloning of TMEM16A as a calcium-activated chloride channel subunit. Cell.

[13] Miledi R (1982). Calcium-dependent transient outward current in *Xenopus laevis* oocytes. Proc. R. Soc. Lond. B. Biol. Sci..

[14] Liu G, Cheresh P, Kamp DW (2013). Molecular basis of asbestos-induced lung disease. Annu. Rev. Pathol..

[15] Kamp DW, Graceffa P, Pryor WA, Weitzman SA (1992). The role of free radicals in asbestos-induced diseases. Free Radic. Biol. Med..

[16] Gazzano E (2007). Iron-loaded synthetic chrysotile:  a new model solid for studying the role of iron in asbestos toxicity. Chem. Res. Toxicol..

[17] Warheit DB (1994). A review of some biophysical factors and their potential roles in the development of fiber toxicity. Regul. Toxicol. Pharmacol..

[18] Hardy JA, Aust AE (1995). Iron in asbestos chemistry and carcinogenicity. Chem. Rev..

[19] Weitzman SA, Graceffa P (1984). Asbestos catalyzes hydroxyl and superoxide radical generation from hydrogen peroxide. Arch. Biochem. Biophys..

[20] Aust AE, Cook PM, Dodson RF (2011). Morphological and chemical mechanisms of elongated mineral particle toxicities. J. Toxicol. Environ. Health B. Crit. Rev..

[21] Klebanoff SJ, Foerder CA, Eddy EM, Shapiro BM (1979). Metabolic similarities between fertilization and phagocytosis. Conservation of a peroxidatic mechanism. J. Exp. Med..

[22] Lim JB, Langford TF, Huang BK, Deen WM, Sikes HD (2016). A reaction-diffusion model of cytosolic hydrogen peroxide. Free Radic. Biol. Med..

[23] Appenzeller-Herzog C (2016). Transit of H_2_O_2_ across the endoplasmic reticulum membrane is not sluggish. Free Radic. Biol. Med..

[24] Borelli, V., Trevisan, E., Vita, F. & Zabucchi, G. The secretory response of rat peritoneal mast cells on exposure to mineral fibers. *Int*. *J*. *Environ*. *Res*. *Public Health* **15**, 10.3390/ijerph15010104 (2018). 10.3390/ijerph15010104 PMC5800203 29320402

[25] Popescu BF, Belak ZR, Ignatyev K, Ovsenek N, Nichol H (2007). Asymmetric distribution of metals in the *Xenopus laevis* oocyte: a synchrotron X-ray fluorescence microprobe study. Biochem. Cell Biol..

[26] Kandror KV, Tsuprun VL, Stepanov AS (1992). The main adenosine triphosphate-binding component of amphibian oocytes is ferritin. Mol. Reprod. Dev..

[27] Huang WH, Guo HB, Huang XY, Sun FZ (2003). Two types of new ferritin cDNA sequences from *Xenopus Laevis* germinal vesicle oocytes. DNA Seq..

[28] Zanella D (2017). Iron oxide nanoparticles can cross plasma membranes. Sci. Rep..

[29] Andolfi L (2013). The crocidolite fibres interaction with human mesothelial cells as investigated by combining electron microscopy, atomic force and scanning near-field optical microscopy. J. Microsc..

[30] Mandato CA, Bement WM (2001). Contraction and polymerization cooperate to assemble and close actomyosin rings around *Xenopus* oocyte wounds. J. Cell Biol..

[31] Kourie JI (1998). Interaction of reactive oxygen species with ion transport mechanisms. Am. J. Physiol..

[32] Stark G (2005). Functional consequences of oxidative membrane damage. J. Membr. Biol..

[33] Duprat F (1995). Susceptibility of cloned K+channels to reactive oxygen species. PNAS.

[34] Cougnon M, Benammou S, Brouillard F, Hulin P, Planelles G (2002). Effect of reactive oxygen species on NH^4+^ permeation in *Xenopus laevis* oocytes. Am. J. Physiol. Cell Physiol..

[35] Schlief T, Heinemann SH (1995). H_2_O_2_-induced chloride currents are indicative of an endogenous Na^+^-Ca^2+^ exchange mechanism in *Xenopus* oocytes. J. Physiol..

[36] Kim MJ, Han JK (2002). Hydrogen peroxide-induced current in *Xenopus* oocytes: current characteristics similar to those induced by the removal of extracellular calcium. Biochem. Pharmacol..

[37] Miledi R, Parker I, Sumikawa K (1987). Oscillatory chloride current evoked by temperature jumps during muscarinic and serotonergic activation in *Xenopus* oocyte. J. Physiol..

[38] Barish ME (1983). A transient calcium-dependent chloride current in the immature *Xenopus* oocyte. J Physiol..

[39] Schreiber R (2018). Regulation of TMEM16A/ANO1 and TMEM16F/ANO6 ion currents and phospholipid scrambling by Ca^2+^ and plasma membrane lipid. J. Physiol..

[40] Ghio AJ, Taylor DE, Stonehuerner JG, Piantadosi CA, Crumbliss AL (1998). The release of iron from different asbestos structures by hydrogen peroxide with concomitant O_2_ generation. Biometals.

[41] Yoon JH (2011). Oxidative modification of ferritin induced by hydrogen peroxide. BMB Rep..

[42] Turci F, Tomatis M, Lesci IG, Roveri N, Fubini B (2011). The iron-related molecular toxicity mechanism of synthetic asbestos nanofibres: a model study for high-aspect-ratio nanoparticles. Chemistry.

[43] Valko M, Jomova K, Rhodes CJ, Kuča K, Musílek K (2016). Redox- and non-redox-metal-induced formation of free radicals and their role in human disease. Arch. Toxicol..

[44] Montesano L, Carrì MT, Mariottini P, Amaldi F, Rotilio G (1989). Developmental expression of Cu,Zn superoxide dismutase in. Xenopus. Eur. J. Biochem..

[45] Fang R, Aust AE (1997). Induction of ferritin synthesis in human lung epithelial cells treated with crocidolite asbestos. Arch. Biochem. Biophys..

[46] Aung W, Hasegawa S, Furukawa T, Saga T (2007). Potential role of ferritin heavy chain in oxidative stress and apoptosis in human mesothelial and mesothelioma cells: implications for asbestos-induced oncogenesis. Carcinogenesis.

[47] Ghio AJ, LeFurgey A, Roggli VL (1997). *In vivo* accumulation of iron on crocidolite is associated with decrements in oxidant generation by the fiber. J. Toxicol. Environ. Health.

[48] Kohyama N, Shinohara Y, Suzuki Y (1996). Mineral phases and some reexamined characteristics of the international union against cancer standard asbestos samples. Am. J. Ind. Med..

[49] Bernareggi A, Reyes-Ruiz JM, Lorenzon P, Ruzzier F, Miledi R (2011). Microtransplantation of acetylcholine receptors from normal or denervated rat skeletal muscles to frog oocytes. J. Physiol..

[50] Englund UH, Gertow J, Kågedal K, Elinder F (2014). A Voltage dependent non-inactivating Na+channel activated during apoptosis in *Xenopus* oocytes. PLoS One.

[51] Guilbault GG, Kramer DN, Hackley EB (1967). New substrate for fluorometric determination of oxidative enzymes. Anal. Chem..

[52] Bellavite P, Dri P, Bisiacchi B, Patriarca P (1977). Catalase deficiency in myeloperoxidase deficient polymorphonuclear leucocytes from chicken. FEBS.

