# Supplementary material for: On the mechanism of the electrophysiological changes and membrane lesions induced by asbestos fiber exposure in Xenopus laevis oocytes
Source: Sci Rep. 2019 Feb 14;9:2014. doi: 10.1038/s41598-019-38591-x (PMC6376119; doi:10.1038/s41598-019-38591-x)
Supplement: Supplementary file 1 — Supplementary info [file 41598_2019_38591_MOESM1_ESM.docx]

**Supplementary informations:**

**On the mechanism of the electrophysiological changes and membrane lesions induced by asbestos fiber exposure in *Xenopus laevis* oocytes**

**Authors:** Annalisa Bernareggi^1*^, Giorgia Conte^1^, Andrew Constanti^2^, Violetta Borelli^3^, Francesca Vita^3^, and Giuliano Zabucchi^3*^

**Affiliations:**

^1^ Department of Life Sciences and Centre for Neuroscience B.R.A.I.N., University of Trieste, via Fleming 22, 34127, Trieste, Italy

^2^Department of Pharmacology, UCL School of Pharmacy, 29/39 Brunswick Square, London, WC1N 1AX, United Kingdom

^3^Department of Life Sciences, University of Trieste, University of Trieste, via Valerio 28/1, 34127, Trieste, Italy

*** Corresponding authors:**

Prof. Annalisa Bernareggi

[abernareggi@units.it](mailto:abernareggi@units.it)

Phone number: +39 040 558 8619

Prof. Giuliano Zabucchi

e-mail: [zabucchi@units.it](mailto:abernareggi@units.it)

Phone number: +39 040 558 8660


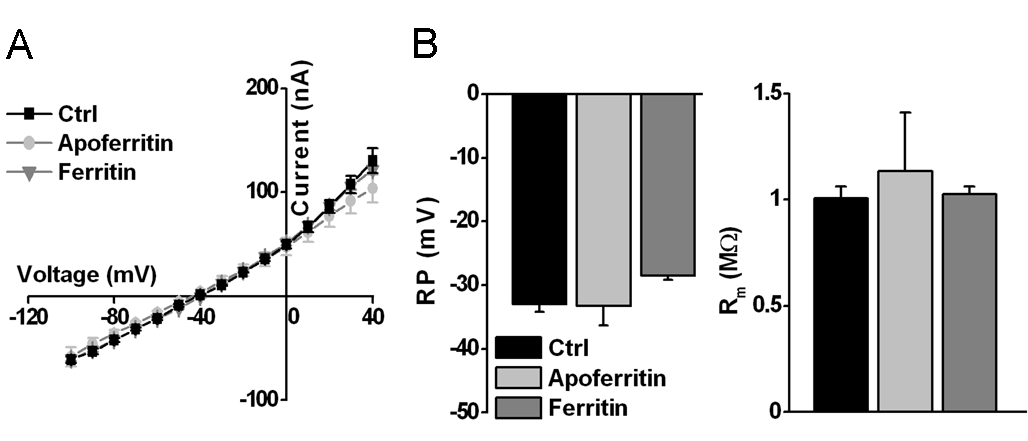


**Supplementary figure 1.** **Test of apoferritin (500 ng/ml) and ferritin (~4,500 Fe^3+^ ions/molecule, 500 ng/ml) as alternative sources of iron on electrical membrane properties of *Xenopus* oocytes.** The treatment with apoferritin or ferritin (5-30 minutes) did not affect the *I-V* relationships or the passive membrane properties of the oocytes (Ctrl, *n* = 5; apoferritin, *n* = 4; ferritin; *n* = 4, oocytes from the same donor). V_h_ = -40 mV, voltage steps: -100 mV to +40 mV, 10 mV intervals. Values expressed as Mean ± SEM.


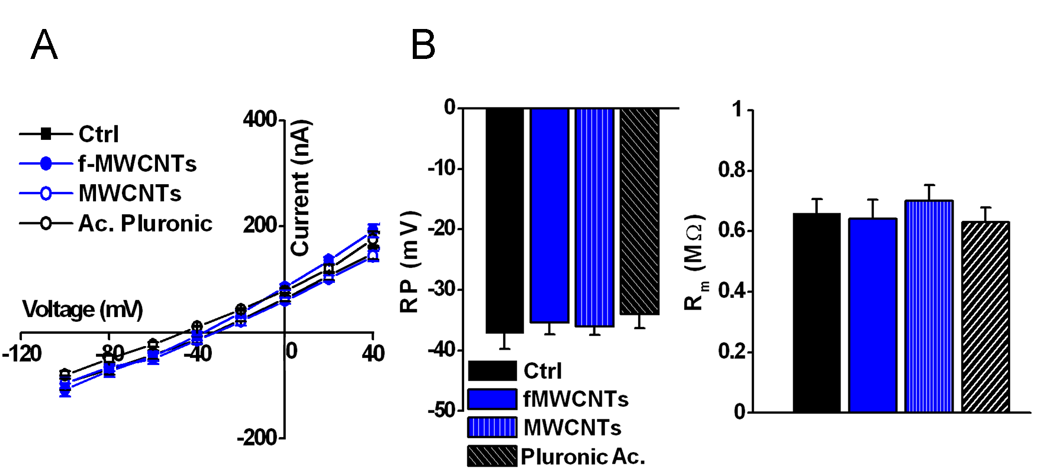


**Supplementary figure 2.** **Test of carbon nanotube fibers (f-MWCNT and MWCNT) on electrical membrane properties of *Xenopus* oocytes.** The cells were incubated in the presence of 15-45 μg/ml f-MWCNT or MWCNT for 5-30 minutes. The f-MWCNT were functionalized in the presence of pluronic acid (Ctrl *n* = 17, f-MWCNT *n* = 18; MWCNT *n* = 9, pluronic acid *n* = 5). In (*A*), the *I-V* relationships, in (*B*) the comparison of the RP and R_m_ values of in the same oocytes. The treatments did not affect the *I-V* relationships or the passive membrane properties of the oocytes. V_h_ = -40 mV, voltage steps: -100 mV to +40 mV, 20 mV intervals. Values expressed as Mean ± SEM.


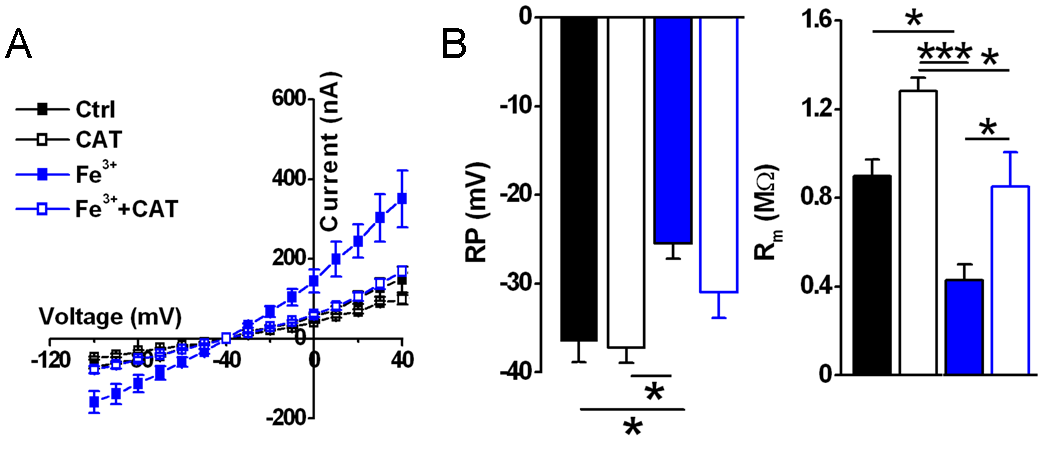


Supplementary figure 3. Catalase (CAT) prevents the effect induced by Fe^3+^_._ Oocytes were treated with Fe^3+^ (400 μM, *n* = 4) for 5-30 minutes, CAT (250 U/ml, *n* = 4) and co-treated in the presence of Fe^3+^ (400 μM) and CAT (250 U/ml) for 5-30 minutes (*n* =4). In (*A*), the *I-V* relationships, in (*B*) the comparison of the RP and R_m_ values of in the same oocytes. Oocytes from same donor (Ctrl *n* = 4). V_h_ = -40 mV, voltage steps: -100 mV to +40 mV, 10 mV intervals. Mean ± SEM, **P* < 0.05, ***P* < 0.01, ****P* < 0.001. One-Way Anova test (with *Tukey post hoc*). Oocytes from the same donor.


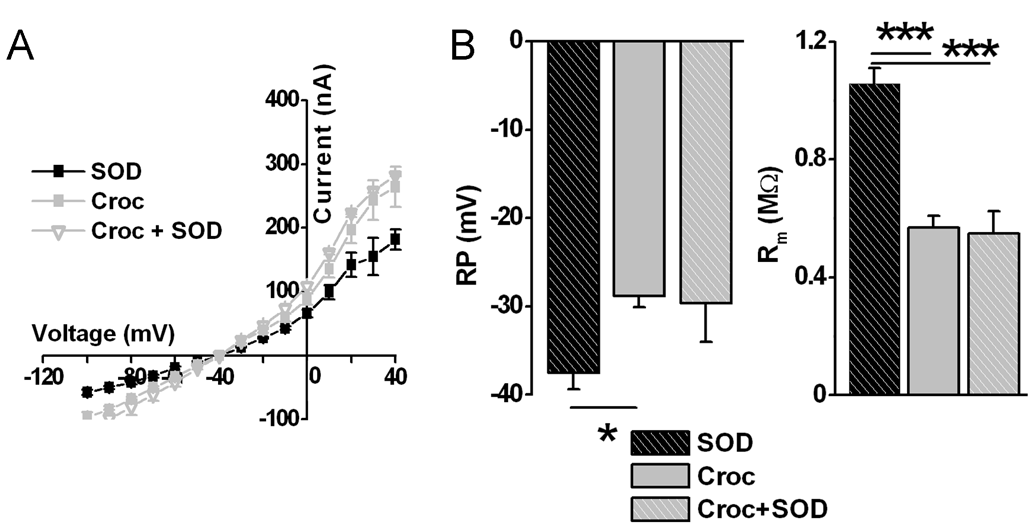


# Supplementary figure 4. Superoxide dismutase (SOD) does not prevent the effect mediated by crocidolite on the oocyte membrane. Oocytes from the same donor were treated in the presence of SOD (5 μg/ml, *n* = 4), crocidolite (*n* = 5) and Croc + SOD (*n* = 3). (*A*) Comparison of the *I-V* relationships and (*B*) RP and R_m_. V_h_ = -40 mV, voltage steps: -100 mV to +40 mV, 10 mV intervals. Mean ± SEM. **P* < 0.05, ***P < 0.001*.* One-Way Anova test (with *Tukey’s post hoc*).
